# Geostatistical modelling of malaria indicator survey data to assess the effects of interventions on the geographical distribution of malaria prevalence in children less than 5 years in Uganda

**DOI:** 10.1371/journal.pone.0174948

**Published:** 2017-04-04

**Authors:** Julius Ssempiira, Betty Nambuusi, John Kissa, Bosco Agaba, Fredrick Makumbi, Simon Kasasa, Penelope Vounatsou

**Affiliations:** 1 Swiss Tropical and Public Health Institute, Basel, Switzerland; 2 University of Basel, Basel, Switzerland; 3 School of Public Health, Makerere University, Kampala, Uganda; 4 Ministry of Health, Kampala, Uganda; Universidade Federal de Minas Gerais, BRAZIL

## Abstract

**Background:**

Malaria burden in Uganda has declined disproportionately among regions despite overall high intervention coverage across all regions**.** The Uganda Malaria Indicator Survey (MIS) 2014–15 was the second nationally representative survey conducted to provide estimates of malaria prevalence among children less than 5 years, and to track the progress of control interventions in the country. In this present study, 2014–15 MIS data were analysed to assess intervention effects on malaria prevalence in Uganda among children less than 5 years, assess intervention effects at regional level, and estimate geographical distribution of malaria prevalence in the country.

**Methods:**

Bayesian geostatistical models with spatially varying coefficients were used to determine the effect of interventions on malaria prevalence at national and regional levels. Spike-and-slab variable selection was used to identify the most important predictors and forms. Bayesian kriging was used to predict malaria prevalence at unsampled locations.

**Results:**

Indoor Residual Spraying (IRS) and Insecticide Treated Nets (ITN) ownership had a significant but varying protective effect on malaria prevalence. However, no effect was observed for Artemisinin Combination-based Therapies (ACTs). Environmental factors, namely, land cover, rainfall, day and night land surface temperature, and area type were significantly associated with malaria prevalence. Malaria prevalence was higher in rural areas, increased with the child’s age, and decreased with higher household socioeconomic status and higher level of mother’s education. The highest prevalence of malaria in children less than 5 years was predicted for regions of East Central, North East and West Nile, whereas the lowest was predicted in Kampala and South Western regions, and in the mountainous areas in Mid-Western and Mid-Eastern regions.

**Conclusions:**

IRS and ITN ownership are important interventions against malaria prevalence in children less than 5 years in Uganda. The varying effects of the interventions calls for selective implementation of control tools suitable to regional ecological settings. To further reduce malaria burden and sustain malaria control in Uganda, current tools should be supplemented by health system strengthening, and socio-economic development.

## Background

Malaria remains one of the leading public health burdens in the world despite the remarkable achievements made towards its control and prevention since the beginning of the second millennium. Recent global estimates indicate that malaria is responsible for over 214 million cases and over 438,000 deaths [1]. Most of this burden is concentrated in Sub-Saharan Africa (SSA) region which accounts for 90% of the mortality burden, most of which occur among children less than 5 years old [1]. However, malaria has gone down from first to fourth highest cause of mortality in this age group during the last 15 years [1].

Uganda has the fourth highest number of *Plasmodium falciparum* infections [1] and some of the highest reported malaria transmission rates in the world [2]. Ninety five percent of the country has stable malaria transmission, with the rest having low and unstable transmission with potential for epidemics. Malaria is responsible for 33% of all outpatient visits and 30% of hospital admissions [3]. Ninety-nine percent of malaria cases are attributed to *P*. *falciparum* species—*Anopheles gambiae* s.1 and *An*. *funestus* being the most common vectors [4].

Vector control tools, that is, Insecticide Treated Nets (ITNs), Indoor Residual Spraying (IRS), and case management with Artemisinin-based Combination Therapies (ACTs) are at the forefront of malaria control and prevention in Uganda [3]. Malaria Indicator Surveys (MIS) are nationally representative surveys conducted every 5 years to estimate malaria prevalence among children of age less than 5 years and track the progress of coverage of control interventions. The most recent MIS conducted in Uganda showed that overall prevalence of malaria among children age less than 5 years was 19.0% [5]. Results also indicated that coverage of interventions was high across all regions. However, there were wide variations in regional malaria prevalence, varying from less than 5% in Kampala and South Western regions to over 25% in East Central, North East and West Nile regions [5]. Whether the differences in the prevalence are due to variations in climatic, socio-economic, and demographic characteristics, or as a result of intervention effects varying in space needs to be investigated empirically.

MIS have been used to analyse the effect of interventions on malaria prevalence using both non-spatial and bayesian geostatistical methods. The latter give reliable estimates because they take into account correlation of malaria prevalence in space arising from common exposures affecting neighbouring areas similarly. Bayesian geostatistical models have been used in mapping of malaria burden [6] and recently in the analysis of MIS data in high endemic countries of SSA, namely, Zambia [7], Angola [8], Tanzania [9], Senegal [10], Nigeria [11] and Burkina Faso [12]. Despite comparable malaria transmission intensities in these countries, findings showed varied effects of interventions on malaria prevalence among children less than 5 years. For instance, a protective and non-protective effects were reported for ITNs and IRS respectively in Zambia [7], Angola [8] and Senegal [10]. On the other hand, no effects were observed for the role of interventions in Nigeria [11], and Tanzania [9]. In Liberia [13] and Burkina Faso [12], intervention effects were protective at sub-national level but had no effect at country level.

In the current study, we analysed the Uganda MIS 2014–15 using bayesian geostatistical models to: i) determine the effect of interventions on malaria prevalence in children less than 5 years adjusted for environmental, demographic and socio-economic characteristics, ii) assess intervention effects at regional level, and iii) obtain spatially explicit estimates of malaria prevalence in this age group. A malaria risk map is a vital tool for efficient planning, resource mobilisation, monitoring and evaluation. To date, the only map available for Uganda is the one extracted from the new world malaria map [6] which is now out-dated since it does not take into account contemporary effects of interventions, socio-economic status and climatic/environmental conditions.

## Methods

### Country profile

Uganda is a land locked country located in East Africa, and shares borders with South Sudan to the north, Kenya to the east, Democratic Republic of Congo to the west, and Tanzania and Rwanda to the south. It lies between latitudes 1^0^ south and 4^0^ north of the equator, with altitude ranging from 620 m to 5,111 m above sea level, and mean annual temperatures between 14°C and 32°C. It has two rainfall seasons in a year, a shorter one during March to May and a longer season spanning September to December. A range of ecosystems cover the country with the south dominated by tropical rain forests which gradually turn into savannah woodland and semi-desert in the north. The country is divided into 112 districts grouped into 10 regions and covers an area of about 241,039 square kilometres.

Uganda has a population of 35 million people living in 7.3 million households [14]. The population is largely young with 50% of the population constituted with individuals of age 0–15 years. The proportion of population of children age less than 5 years is 17.7% [14].

### Uganda MIS 2014–15

The 2014–15 MIS was based on a stratified two-stage cluster design [5]. In the first stage, 20 sampling strata were created and 210 clusters were selected with probability- proportional-to-size sampling. At the second stage, using complete lists of households in the selected clusters, 28 households were chosen from each cluster with equal probability systematic sampling.

All women of age 15–49 years in the sampled households, who were either permanent residents or visitors in the household on the night preceding the survey, were eligible for interview. Similarly, all children of age less than 5 years were eligible for malaria testing.

Blood samples were taken from fingers or heels of children age less than 5 years and tested on-spot using Rapid Diagnostic Tests (RDTs). In addition, thick and thin blood smears were prepared and tested by microscopy. Results were recorded as either positive or negative if malaria parasites were found or not in the blood sample, respectively. In this study, microscopy results were considered because of the reduced sensitivity of RDTs in populations that have recently been treated and cleared of malaria parasites due to the presence of the residual HRP2 antigen [15].

### Ethical approval

In this study we used secondary data that was made available by the Uganda Bureau of Statistics (UBOS) and the Demographic Health Survey (DHS) MEASURE group based in the United States of America. According to survey protocols and related documents [5], the ethical approval process was described as follows; The Institutional Review Board of International Consulting Firm (ICF) of Calverton, Maryland, USA reviewed and approved the Uganda MIS 2014–15. This complied with the United States Department of Health and Human Services requirements for the "Protection of Human Subjects" (45 CFR (Code of Federal regulations) 46).

The survey was also reviewed and approved by Makerere University School of Biomedical Sciences Higher Degrees Research and Ethics committee (SBS-HDREC), and the Uganda National Council for Science and Technology (UNCST).

An interview was conducted only if the respondent provided their verbal consent in response to being read an informed consent statement by the interviewer. Also, verbal informed consent for each parasitaemia test was provided by the child’s parent/guardian/caregiver on behalf of children less than 5 years before the test was conducted. Verbal consent was conducted by the interviewer reading a prescribed statement to the respondent and recording in the questionnaire whether or not the respondent consented or assent was provided. The interviewer signed his or her name attesting to the fact that he/she read the consent statement to the respondent. Verbal consent was preferred over written consent because of low literacy levels especially in rural areas of Uganda [5]

### Predictor variables

Malaria transmission is known to be influenced by several factors including interventions [16], environmental/climatic [17], socio-economic [18] and demographic factors [19]. Environmental/climatic proxy variables were extracted from remote sensing sources for the period February 2014 –January 2015 (Table 1).

**Table 1:** Sources, spatial and temporal resolution of environmental/climatic and population data.

Data	Source	Period	Spatial resolution	Temporal resolution
Annual average Day and Night Land Surface Temperature (LST)	MODIS	February 2014- January 2015	1x1km^2^	8 days
Annual average Normalized Difference Vegetation Index (NDVI)	MODIS	February 2014- January 2015	1x1km^2^	16 days
Population data	Worldpop	2014	0.1x0.1km^2^	na
Annual average Rainfall	U.S. Geological Survey-Earth Resources Observation Systems (USGSS)	February 2014- January 2015	8x8km^2^	10 days
Altitude (Digital Elevation Model)	Shuttle Radar Topographic Mission (SRTM)	2000	0.5x0.5km^2^	na
Water bodies	MODIS	-	0.5x0.5km^2^	na
Urban Rural extent	Global Rural and Urban Mapping project	February 2014- January 2015	1x1km^2^	na

MODIS: Moderate Resolution Imaging Spectroradiometer

na: Not applicable

Demographic variables were captured on survey tools, namely, age of the child, residential location of the household, and mother’s highest level of education.

Data on control interventions were captured on survey questionnaires including ownership and use of ITNs, ACT use and IRS. The data on IRS coverage were collected at household level, whereas that of ITN and ACT use was collected for each child in the selected household. Intervention coverage indicators were generated following standard definitions of Roll Back Malaria [20]. The ITN ownership indicators generated and used in the study were; proportion of households with at least one ITN (pro_1ITN), proportion of households with one ITN for every two people (pro_1ITN4two), and proportion of population with access to an ITN within their household (pro_itnaccess). ITN use indicators were; proportion of children less than 5 years who slept under an ITN on the night preceding the survey (pro_slept5itn), proportion of population that slept under an ITN in the night preceding the survey (pro_sleptitn), and proportion of ITNs used last night preceding the survey (pro_itnused).

ACT coverage was measured as the proportion of fevers reported in the last 2 weeks before the survey that were treated with any ACTs. The indicator on IRS coverage was derived as the proportion of households sprayed in the last 6 months.

The wealth index available in the data and calculated as a weighted sum of household assets using principal component analysis [21] was used a proxy for socio-economic status.

Prior to bayesian model fitting, collinearity between all pairs of independent variables was assessed using non-spatial regression methods based on values of Variance Inflation Factor (VIF) and Tolerance Values (TR).

### Bayesian geostatistical modelling

Three bayesian geostatistical logistic regression models were fitted to determine the geographical distribution of malaria prevalence in children less than 5 years in Uganda, assess the adjusted effect of interventions on malaria prevalence, and estimate the effects of interventions at regional level. The first model included only environmental predictors, the second comprised of environmental, demographic, and socio-economic factors, whereas the third was modelled with spatially varying coefficients for interventions adjusted for the effect of environmental, socio-economic status and demographic predictors. The third model assesses the effects of interventions at regional level using spatially varying coefficients [13] and is formulated assuming a conditional autoregressive (CAR) prior distribution [22] which introduces a neighbour-based spatial structure for the regression coefficients for each intervention effect [23]. Neighbours were defined as the adjacent areas for each region. This model was adjusted for the effect of environmental/climatic, socio-economic status and demographic factors.

The outcome of interest was the parasitaemia test result of a child tested in a sampled household.

To adjust for spatial correlation present in malaria data due to similar exposure effect in neighbouring areas, cluster-specific random effects were added to each model. The cluster random effects were assumed to arise from a Gaussian stationary process with a covariance matrix capturing correlation between any pair of cluster locations as a function of their distances.

To improve model fit and parameter estimation, bayesian geostatistical variable selection was used to select the most important predictors and form in explaining variation in malaria prevalence for the three models mentioned above. In model 1, selection consisted of introducing an indicator variable for every climatic predictor and estimating the probabilities of excluding or including the predictor into the model in linear or categorical form. These probabilities indicate the proportion of models including a given predictor out of models generated from all combinations of predictors. Variables were categorized using predictor quartiles. Only variables with an inclusion probability of more than 50% were used to predict malaria prevalence in children less than 5 years at unsampled locations.

Similarly in the second model, geostatistical variable selection was performed to choose the most important intervention, socio-economic and demographic predictors for malaria prevalence. This model was adjusted for the effect of environmental predictors fitted in model 1. The indicator with the highest probability of inclusion per group of ITN ownership (pro_1ITN, pro_1ITN4two, pro_itnaccess) and ITN use (pro_slept5itn, pro_sleptitn, pro_itnused) was selected.

Prediction of malaria prevalence was performed using bayesian kriging [24] over a regular grid of 52,495 pixels at 4 km^2^ resolution covering the entire country.

The population-adjusted number of individuals infected with malaria was estimated by first combining the high spatial resolution population data obtained from worldpop [25] with the predicted pixel-level malaria prevalence estimates. The population data were re-scaled from their initial 100x100m spatial resolution to the 2x2km resolution of the gridded risk estimates. The number of children less than 5 years infected with malaria per pixel was estimated by multiplying population counts by a factor of 17.7%—the proportion of population under 5 years [14]. The pixel-level estimates were aggregated at regional level to produce number infected per region.

Data analysis was carried out in STATA (StataCorp. 2015. *Stata Statistical Software*: *Release 14*. College Station, TX: StataCorp LP). OpenBUGS version 3.2.3 (Imperial College and Medical Research Council, London, UK) was used to implement the variable selection approach and to perform model fit. The bayesian kriging was implemented using a program written by the authors in R statistical computing and graphics software [26]. Maps were produced using ESRI’s ArcGIS 10.2.1 for Desktop (http://www.esri.com/).

Parameter estimates were summarized using posterior medians and the corresponding 95% Bayesian Credible Intervals (BCI). Model estimates were exponentiated to produce Odds Ratios (OR). The effect of a predictor was considered to be important if the 95%BCI of the coefficient did not include a zero. The details of the fitted models are given in the S1 Text.

## Results

**Fig 1:**
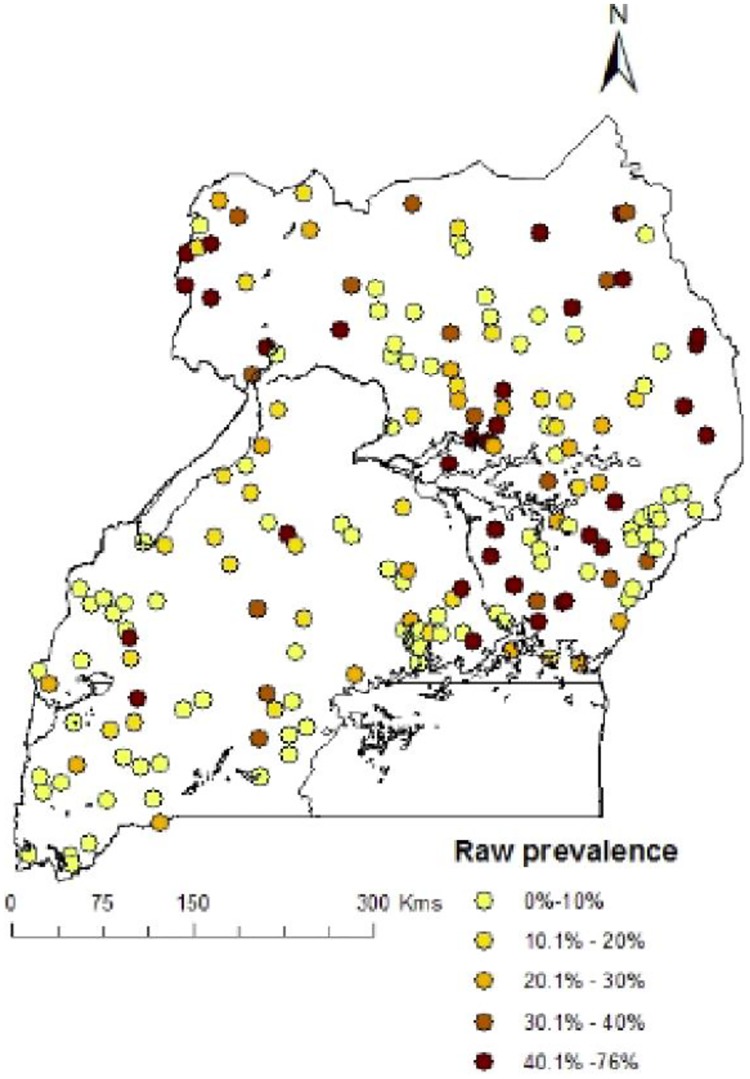
Observed malaria prevalence at survey locations in Uganda, MIS 2014–15.

A total of 4939 children age 0–59 months were tested for malaria from 210 clusters. The overall prevalence of malaria by microscopy was 19.0%. However, in this study we used data from only 193(91.9%) clusters whose geo-referenced information was available at the time of analysis (Fig 1). This reduced sample had 4591 children tested for malaria with malaria prevalence of 19.5% which varied from 0% in Kampala region to over 38.0% in East Central region. Table 2 shows the overall and regional coverage distribution of intervention indicators.

**Table 2:** Coverage of control interventions by region.

Region	Number of Clusters	Prevalence	pro_1ITN^a^	pro_1ITN4two^b^	pro_slept5itn^c^	IRS^d^	ACT^e^	pro_itnaccess^f^	pro_sleptitn^g^
North East	32	32.3	0.96	0.51	0.86	0.02	0.71	0.80	0.84
West Nile	16	27.4	0.96	0.64	0.76	0.02	0.65	0.86	0.79
Mid-North	31	14.8	0.94	0.54	0.77	0.55	0.71.	0.82	0.77
Mid-Western	14	14.1	0.96	0.52	0.83	0.0	0.64	0.81	0.80
Mid-Eastern	24	14.1	0.97	0.52	0.81	0.0	0.79	0.82	0.76
East Central	15	38.6	0.86	0.36	0.69	0.0	0.72	0.67	0.66
Central 2	17	20.4	0.89	0.46	0.66	0.0	0.72	0.73	0.66
Central 1	17	11.2	0.87	0.51	0.65	0.02.	0.60	0.74	0.62
South Western	11	4.5	1.00	0.69	0.66	0.0	0.60	0.91	0.67
Kampala	16	0.0	0.94	0.62	0.71	0.03	0.57	0.82	0.77
Overall	193	19.5	0.94	0.54	0.76	0.1	0.68	0.81	0.75

^a^Proportion of households with at least one ITN

^b^Proportion of households with at least one ITN for every two people

^c^Proportion of children less than 5 years who slept under an ITN

^d^Proportion of households sprayed in the last 6 months

^e^Proportion of fevers treated with any ACTs

^f^Proportion of population who had access to an ITN

^g^Proportion of population who slept under an ITN

Nine out of every ten households had an ITN, but the proportion of households having one ITN for every two people was lower, varying from 36.3% in East Central to almost 70% in South Western region. At country level, 80% of the population had access to an ITN in their households, with coverage ranging from 67% in East Central region to over 90% in South Western region. Seventy-five percent of the population slept under an ITN on the night preceding the survey. Comparing ITN assess and ITN use show a surplus of 5% unused ITNs.

Three out of every four children of age less than 5 years slept under an ITN—the lowest coverage was observed in Central 1 region, while the highest was reported in North East region.

Case management using ACTs ranged from 60% in Kampala to almost 80% in East Central region.

About one out of every ten households in the country had been sprayed in the last 6 months, but this intervention was mainly implemented in the Mid-North region where almost 6 out of every 10 households were sprayed.

In Table 3, results from the bayesian geostatistical variable selection are presented. In model 1, day LST (categorical), night LST (linear), land cover and area type were selected. These selected variables were used for predicting malaria prevalence in children less than 5 years at unsampled locations. Results in model 2 indicate a high probability of inclusion (>90%) for IRS, wealth index, age and mother’s highest level of education. However, indicators for ITN and ACTs were selected with low probabilities which might be indicative of a weak relationship with malaria prevalence.

**Table 3:** Posterior inclusion probabilities for environmental, intervention, socio-economic and demographic factors.

Variable	Posterior inclusion probability (%)	
	Model 1^†^	Model 2^††^
Land surface temperature (day)	0.0	0.0
Land surface temperature (night)	87.6	74.1
Normalized difference vegetation index	41.9	36.2
Rainfall	4.6	26.9
Altitude	12.6	27.2
Distance to water bodies	0.0	14.1
Land cover	100	100
Land surface temperature (day)*	100	100
Land surface temperature (night)*	0.0	0.0
Normalized difference vegetation index*	0.0	0.0
Rainfall *	0.0	0.0
Altitude *	0.0	0.0
Distance to water bodies*	0.0	0.0
Area type (rural vs urban) *	100	100
***Intervention***		
IRS use		100
***ITN ownership***		
pro_1ITN4two		8.3
pro_1ITN		2.3
pro_itnaccess		27.4
***ITN use***		
pro_slept5itn		14.3
pro_sleptitn		0.0
pro_itnused		17.6
***Case management of malaria at health facilities***		
Proportion of fevers treated with any anti-malarial		12.9
Proportion of fevers treated with ACTs		53.6
Socioeconomic status, demographic		
Wealth index		100
Area type		93.7
Age		100
Mother’s highest level of education		94.4

* Categorical form

^†^Only climatic predictors

^††^Intervention + climatic + SES + demographic

Table 4 presents results from Bayesian geostatistical models. In model 1 results show that day LST, night LST, land cover, and area type were significantly associated with malaria prevalence. Also, increases in day and night LST were significantly associated with higher odds of malaria prevalence. Moreover, the odds of malaria prevalence were more than two times higher in cropping areas compared to forested areas (OR = 2.12 95%BCI: 1.25–2.29).

**Table 4:** Posterior estimates for the effect of environmental, intervention, socio-economic factors.

Variable	Parasitaemia prevalence (%)	Model 1^†^	Model 2^††^
		OR (95% BCI)	OR (95% BCI)
***Land cover***			
Forest	17.8	1.0	1.0
Crops	27.2	2.12 (1.25, 2.29)*	1.35 (1.17, 1.42)*
Others	10.0	0.56 (0.39, 0.73)*	0.59 (0.44, 0.71)*
***Land surface temperature (Day)***			
< = 31.4	11.7	1.0	1.0
31.4–33.8	19.7	1.98 (1.69, 2.52)*	2.87 (2.42, 3.08)*
> = 33.8	26.6	3.19 (2.83, 3.85)*	1.98 (1.68, 2.01)*
***Land surface temperature (Night***)	-	1.75 (1.64, 1.82)*	1.25 (1.17, 1.26)*
***Area type***			
Urban	6.0	1.0	1.0
Rural	21.6	6.25 (5.62, 8.60)*	2.06 (1.96, 2.19)*
***Wealth Index***			
Poorest	27.7		1.0
Poorer	21.1		0.86 (0.72, 1.04)
Middle	20.8		0.77 (0.85, 1.15)
Richer	11.9		0.52 (0.42, 0.61)*
Richest	3.3		0.19 (0.14, 0.27)*
***ITN ownership***			
Proportion of population with access to an ITN in their households			0.78 (0.67, 0.89)*
***ITN use***			
Proportion of ITNs used the previous night			1.68 (1.52, 1.77)*
***Indoor Residual Spraying***			
Not sprayed	21.0		1.0
Sprayed	5.0		0.22 (0.14, 0.42)*
***Case management***			
Proportion of fevers treated with ACTs			1.29 (1.00, 1.38)
***Age (months)***			
< = 12	9.6		1.0
13–24	16.1		2.16 (1.85, 2.41)*
25–36	22.5		3.67 (3.08, 4.16)*
37–48	23.1		3.54 (2.83, 3.83)*
49–59	26.0		4.77 (4.47, 5.97)*
***Mother’s education***			
None	26.4		1.0
Primary	17.8		0.85 (0.74, 0.89)*
Post primary	8.2		0.57 (0.57, 0.67)*
***Variances***			
Gaussian process		0.45 (0.40, 0.48)	0.77 (0.62, 0.80)
Range (km)		52.2 (33.9, 69.5)	47.7 (40.7, 56.4)

^†^Only climatic factors

^††^Intervention + climatic + SES + demographic

*Statistically important

The adjusted effects of interventions on malaria prevalence are shown in model 2. The odds of malaria in children who lived in households that had been sprayed were 78% less than those living in unsprayed houses (95%BCI: 58%-86%). ITN access was associated with decreased odds of malaria prevalence. However, results show a risk factor effect for ITN use and no effect for ACTs use.

A decreasing trend of malaria odds with increasing wealth quintile was observed. Malaria odds were 48% (95%BCI: 39%-58%) and 81% (95%BCI: 73%-86%) lower for richer and richest wealth quintile respectively compared to the poorest quintile.

Rural areas had more than two times the prevalence of malaria compared to urban areas (OR = 2.06 95% BCI: 1.96–2.19).

The prevalence of malaria increased with age of a child reaching almost 5 times higher in children age 49–59 months compared to children age < = 12 months (OR = 4.77 95%BCI: 4.47–5.97).

A decreasing trend of malaria prevalence was observed with mother’s highest level of education. Malaria prevalence was 15% (95%BCI: 11%-26%) and 43% (95%BCI: 33%- 43%) lower in children whose mothers had attained primary and post primary education compared to children whose mothers had no education respectively.

Also, results indicate a strong spatial correlation of malaria prevalence of up to 47.7km (Range: 40.7–56.4).

In Table 5 results from the spatially varying coefficient model are presented and indicate that intervention effects varied by region. The effect of ITN ownership was protective in the regions of North East, West Nile and South Western, whereas that of ITN use was protective in Mid-Western. ACT use was protective in Mid-western, North East, and West Nile regions.

**Table 5:** Posterior median and 95% credible intervals for spatially varying effect of interventions on malaria prevalence.

Region	ITN Ownership	ITN Use	ACTs
	OR (95% BCI)	OR (95% BCI)	OR (95% BCI)
Central 1	0.93 (0.69, 1.28)	1.58 (0.85, 1.72)	1.75 (1.42, 2.40)
Central 2	0.93 (0.70, 1.53)	1.09 (0.73, 2.44)	1.52 (1.19, 1.90)
East central	1.50 (0.77, 1.86)	0.94 (0.63, 1.11)	2.11 (1.69, 4.25)
Kampala	1.17 (0.38, 1.25)	1.44 (0.28, 2.26)	1.03 (0.22, 1.67)
Mid-North	1.16 (0.93, 1.41)	0.92 (0.58, 1.38)	0.36 (0.21, 0.71)*
Mid-western	1.02 (0.84, 1.73)	0.92 (0.75, 0.98)*	0.91 (0.85, 1.21)
Mid-eastern	1.09 (1.00, 1.50)	1.13 (0.91, 1.33)	1.39 (0.90, 2.30)
North East	0.85 (0.73, 0.94)*	0.93 (0.66, 1.09)	0.61 (0.46, 0.68)*
South Western	0.87 (0.50, 0.98)*	0.98 (0.77, 2.06)	1.85 (1.07, 2.19)
West Nile	1.44 (1.14, 1.51)	1.01 (0.68, 1.43)	0.45 (0.41, 0.67)*
*Variance*	**Median (95% BCI)**	**Median (95% BCI)**	**Median (95% BCI)**
Spatially varying	3.27 (1.60, 3.92)	2.97 (1.73, 7.61)	1.01 (0.66, 3.22)

*Statistically important and protective

**Fig 2:**
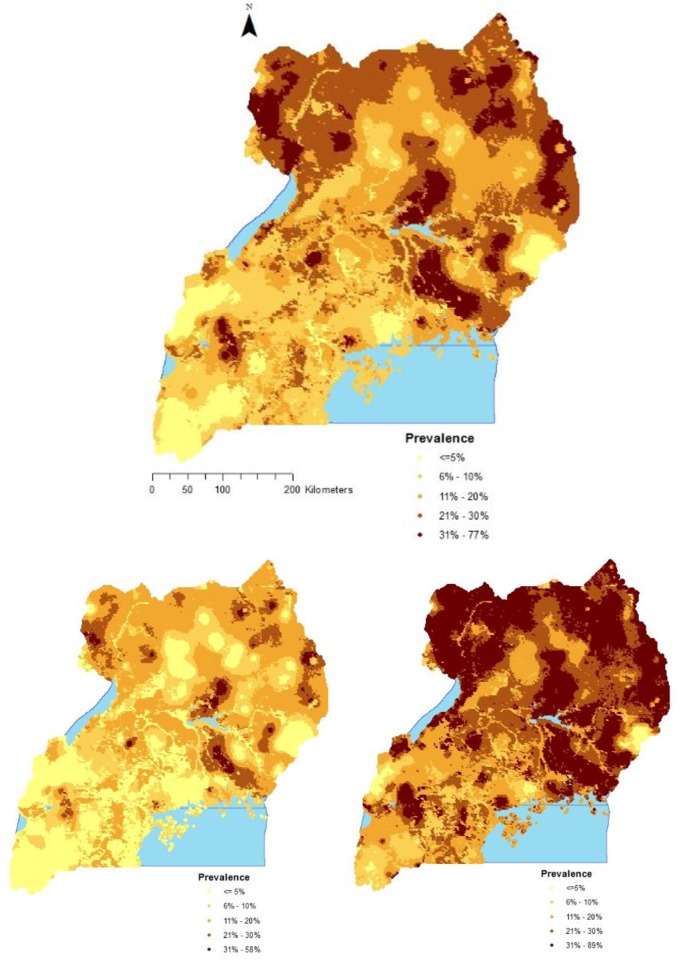
Predicted malaria prevalence in children less than 5 years; median (top), 2.5^th^ percentile (bottom left) and 97.5^th^ percentile posterior predictive distribution (bottom right).

Fig 2 shows maps of the predicted median malaria prevalence, the 2.5^th^ and 97.5^th^ percentiles of the posterior predictive distribution. Malaria prevalence varied from as low as 0.03% to 77.0% with a median of 17.4%. High prevalence (>20.0%) was predicted for regions of East Central, North East, and West Nile, while low prevalence (<5.0%) was predicted for Kampala and South Western regions. More so, a low prevalence was predicted for mountainous areas of Rwenzori and Elgon located in the Mid-Western and Mid-Eastern regions, respectively.

**Fig 3:**
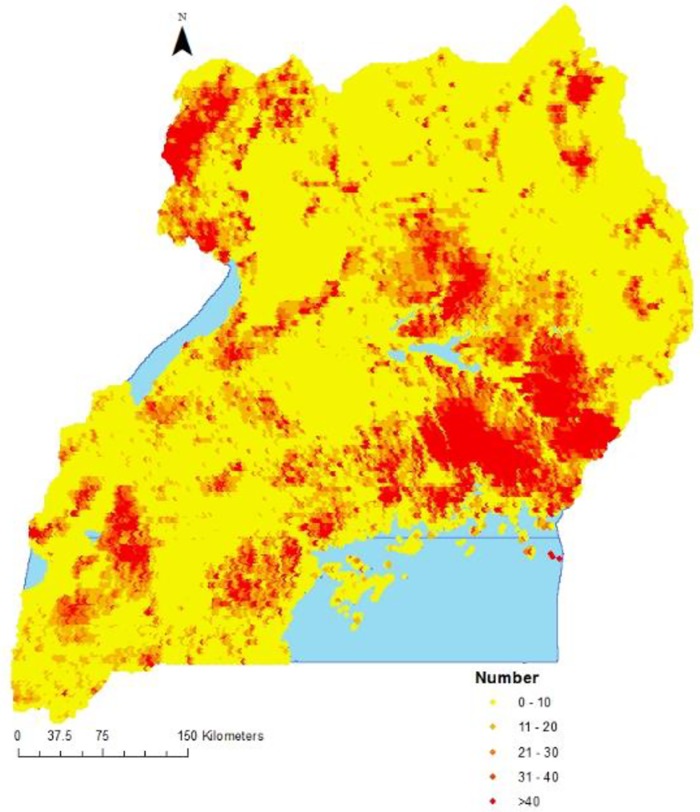
Estimated number of children less than 5 years infected with malaria.

The estimated number of children less than 5 years infected with malaria and the population adjusted prevalences are shown in Table 6. The distribution of infected children in the country is presented in Fig 3.

**Table 6:** Estimated number of infected children less than 5 years and population-adjusted prevalence.

Region	Observed prevalence	Population of under 5 children	Estimated number of infected children	Population adjusted estimated prevalence
	(n/N)		n (95%BCI)	% (95%BCI)
North East	32.3 (277/857)	479,691	119,871 (119,872, 125,485)	23.3 (23.1, 23.4)
West Nile	27.4 (116/423)	414,062	106,377 (100,986, 111769)	25.8 (25.5, 26.0)
Mid-North	14.8 (111/748)	515,113	98,846 (95,745, 101,948)	20.0 (19.8, 20.2)
Mid-Western	14.1 (68/482)	660,687	77,027 (74,023, 80,032)	12.9 (12.7, 13.1)
Mid-Eastern	14.1 (70/498)	524,051	79,734 (76,270, 83,200)	16.8 (16.4, 17.2)
East Central	38.6 (125/324)	516,382	138,191 (132,283, 144,100)	25.3 (24.8, 25.8)
Central 2	20.4 (76/373)	596,969	87,562 (83,516, 91,609)	14.4 (14.2, 14.6)
Central 1	11.2 (34/305)	652,194	58,314 (56,819, 59,208)	10.6 (10.4, 10.8)
South Western	4.5 (19/425)	752,314	56,819 (55,958, 60,671)	8.8 (8.6, 9.1)
Kampala	0.0 (0/156)	357,783	2,895 (2313, 3479)	0.9 (0.8, 1.1)
Overall	19.5 (896/4591)	5,469,245	825,636 (812,316, 838,958)	17.6 (17.1, 17.7)

A total of 825,636 (812,316–839,958) children were estimated to have malaria in 2014. The regions with the highest estimated number of infected children were; East Central, North East and West Nile. Kampala region had the lowest number of infected children. Population adjusted prevalence was 17.6% (95%BCI 17.1%, 17.7%), and varied from 0.9% in Kampala to 26.0% in West Nile. The map shows a highest concentration of infected children in East Central.

## Discussion

In this study we analysed the Uganda 2014–15 MIS data using bayesian geostatistical models to determine the effect of interventions on geographical distribution of malaria prevalence in children less than 5 years in Uganda and its regions, and obtained spatially explicit estimates of malaria prevalence burden in this high risk age group. Indicator variables pertaining to the coverage of interventions of IRS, ITNs, and ACTs were calculated from the data using standard definitions [20].

Bayesian geostatistical models fitted via Markov Chain Monte Carlo simulation methods were used to determine the adjusted effect of interventions on malaria prevalence. Geostatistical variable selection was used to choose the most important predictors for explaining variation in malaria prevalence, and their best functional form to improve model predictive ability and efficiency in parameter estimation.

Land cover, day LST, night LST, and area type were the most important environmental/climatic factors. These variables were among the list of climatic factors compiled in a systematic audit by Weiss et al, 2015 [27] as important for malaria mapping. Also, these findings are similar to results reported from analyses of MIS data in Nigeria and Burkina Faso [11,12].

IRS and ITN ownership had a protective effect against malaria prevalence. Similar results were reported by Roberts and Matthews (2016) [28] who analysed the Uganda MIS 2014–15 data using a classical generalized linear model. The observed strong effect of IRS may be attributed to its effectiveness in killing adult mosquitos as they rest on walls after feeding which cuts short their development cycle and thus reduce vector density resulting in decreased malaria transmission intensity [29]. However, IRS coverage was low in the country with the exception of the Mid-North region where this intervention implemented in 10 districts. Bukirwa et al., (2009) [30] have attributed significant reduction of malaria prevalence, morbidity and mortality in this region to IRS intervention. In other regions, IRS coverage is still very low [5]. The low coverage of this intervention has also been reported in other high endemic countries, namely, Tanzania [9], Burkina Faso [12], Senegal [10], Angola and Mozambique [13]. This could be attributed to the negative campaign against the use of DDT [31].

The protective effect of ITN ownership has been demonstrated in other studies [8,13,32]. However, the observed lower effect of ITNs compared to IRS is inconsistent with results from other studies which showed that ITNs are a more effective and cost-effective tool [33].

Unexpectedly, study results showed an increase of malaria prevalence with ITN use. This finding contradicts findings from other studies that have reported ITN efficacy [32,34,35] and effectiveness [2,16,36,37]. However, these results are consistent to recent findings for Burkina Faso [12], Nigeria [11], Tanzania [9], and Senegal [10]. The lack of protective effect for high ITN use coverage could be attributed to human behaviour such as sleeping patterns where the population tends to stay longer outdoors at night [38], inconsistent ITN use especially during the dry season [39], incorrect use and/or use of worn out ITNs [40], the emerging pyrethroid resistance to insecticides in Uganda [41–43], and high ITN use in areas of high malaria transmission.

Furthermore, results showed a lack of effect of ACTs on malaria prevalence unlike in other studies that demonstrated that ACTs were associated with a reduction in malaria transmission and risk [44,45]. However, this finding should be interpreted cautiously because the data for this intervention was based on reported fevers which had been treated with any ACTs. This unexpected finding may be due to the fact that data for this intervention was based on reported fevers which had been treated with any ACTs. However, no data was available to confirm whether the reported fevers were malaria related or not[5], yet fevers in young children can be caused by several illnesses other than malaria [46]. A similar finding was reported in the Burkina Faso MIS study [12].

Environmental conditions were important predictors of malaria prevalence. This finding further augments the evidence that the environment is a key driver of malaria transmission [47]. Increases in day and night LST were associated with a high malaria prevalence. This relationship can be attributed to the fact that warmer temperatures accelerate larva stages of mosquito lifecycle [48]. Other studies have also arrived at the same conclusion [49].

Areas where crops were grown had higher risk of infection compared to forested areas which may indicate the agricultural transformation effect on the ecological landscape which results in creation of suitable breeding habitats for mosquitoes. Similar results have been reported by Munga et al., (2016) [50].

Living in rural areas was associated with a higher burden of malaria prevalence compared to urban areas. This may be due the fact that rural areas in Uganda are characterized with inadequate health services and poor housing conditions which predisposes individuals to higher malaria prevalence [4,51].

Furthermore, older children were at a higher risk of being infected with malaria compared to infants. This relationship may be due to the fact that infants are partially protected earlier in life by the antibodies from their mothers and passive transfer of the same antibodies through breastfeeding [52,53]. Hendriksen et al., (2013) [54] reported similar findings.

Social economic status was negatively correlated with malaria risk. Children living in wealthier households had a significantly lower malaria risk compared to those living in poorer households. This finding is expected because wealthier people are more likely to afford better health services and afford adequate housing facilities with screens that block mosquitoes resulting in reduced transmission. This finding confirms previous results that showed that malaria burden is highly correlated with poverty [55].

Furthermore, higher mother’s education was associated with reduced malaria prevalence. The role of education in disease prevention cannot be overstated. Highly educated mothers in addition to being more likely to have better socio-economic means, are also mostly likely to have knowledge and means to afford malaria preventive measures. This finding is in agreement with results reported by Fana et al., (2015)[56]. However, mother’s education had no effect on malaria prevalence in Burkina Faso [12].

Results also showed that effects of intervention vary with region—which partially may explain wide variations in malaria prevalence among regions in spite of a high coverage of ITN. Despite the lack of country-level effect for ITN use, the effect of this intervention is significant in Mid-western region. The varying effects of interventions in different regions may be explained by differences in regions with respect to ecological settings, access to health services, and socio-economic development which are important drivers of malaria transmission. Similar findings were reported in Burkina Faso [12], Angola, Liberia, Mozambique, Rwanda, Senegal, and Tanzania [13].

The high malaria prevalence burden predicted for East Central region can be attributed to rice growing [57] which is a predominant economic activity in this region. The rice paddies in which rice is grown serve as suitable habitats for malaria vector breeding. Similarly, the high parasitaemia burden in the North East and West Nile may be due to a very low access to health services [4] and high poverty levels in these regions [51]. On the other hand, a low malaria burden in Kampala region (capital city) can be attributed to better socio-economic conditions [51], reduction in potential mosquito bleeding sites as swamps are reclaimed for residential houses construction [58], and a high access to health services [59]. In South-western region, malaria is low largely due to its location in highlands whose lower temperatures negatively affect vector survival [4].

The risk map illustrates the contemporary malaria situation in the country and can be used for planning, implementation, resource mobilisation, monitoring and evaluation of interventions in the country.

This map differs from that extracted from the 2010 world malaria MAP [6] although outright comparison between these two maps is not possible majorly due to differences in malaria metrics estimated and data sources used. The map from the current study estimates malaria prevalence in the group of children less than 5 years only, whereas the world malaria MAP estimates the burden in the whole population. However, the malaria map produced in this study shows considerable shrinkage in malaria burden in comparison to results from the first MIS survey of 2009 that showed a high burden of malaria in the whole country with the exception of Kampala and highland areas in south western region [60].

There are some limitations of the current study that should be taken into account when interpreting these findings. Firstly, the current study relied on malaria test results from microscopy instead of the gold standard molecular method of polymerase chain reaction (PCR) which is more sensitive than microscopy. Secondly, prediction using spatial methods for data collected from population-weighted sampling designs as the case in MIS may produce imprecise estimates as areas expected to have higher malaria risk are under sampled resulting in higher prediction errors [61].

Furthermore, we did not rescale the varying spatial resolutions of the environmental/climatic remote sensing proxies to a common scale prior to adding them in the models. This may lead to invalid inferences of our study estimates [62].

## Conclusion

This study has demonstrated that IRS and ITN ownership are important interventions against malaria prevalence in children less than 5 years in Uganda, but the effects of all intervention vary by region. Varying intervention effects across regions indicate that interventions do not have a similar effect in different regions. This calls for epidemiological and entomological research in the different settings of the regions to determine the best tools suitable for each region. As well as scaling up of IRS intervention in areas of high transmission and replacing worn-out ITNs with new ones, the government should further strengthen the health system especially in rural areas, embark on socio-economic transformation programs, and introduce new tools such as environmental modification because of the role of these factors on malaria burden in the country.

## Supporting information

S1 Fig Malaria intervention coverage in Uganda in 2014. Top; Prop of HHs with 1 ITN (left), Prop of HHs with 1 ITN for two people (middle), Population with access to an ITN (right), Bottom; Prop who slept under an ITN (left), Prop under 5 slept who under ITN (middle), Prop of fevers treated with ACTs(right)**.**
(PDF)

S2 Fig Distribution of environmental/climatic factors in Uganda in 2014. Top; Altitude (left), Night LST (middle), Day LST (right) Bottom; Rainfall (left), NDVI (middle), Distance to water bodies (right).
(PDF)

S1 Text Statistical modelling details. (DOCX)
