# Supplementary figures and images for: Geostatistical modelling of malaria indicator survey data to assess the effects of interventions on the geographical distribution of malaria prevalence in children less than 5 years in Uganda
Source: PLoS One. 2017 Apr 4;12(4):e0174948. doi: 10.1371/journal.pone.0174948 (PMC5380319; doi:10.1371/journal.pone.0174948)

**S1 Fig: Coverage of malaria intervention indicators in Uganda in 2014**

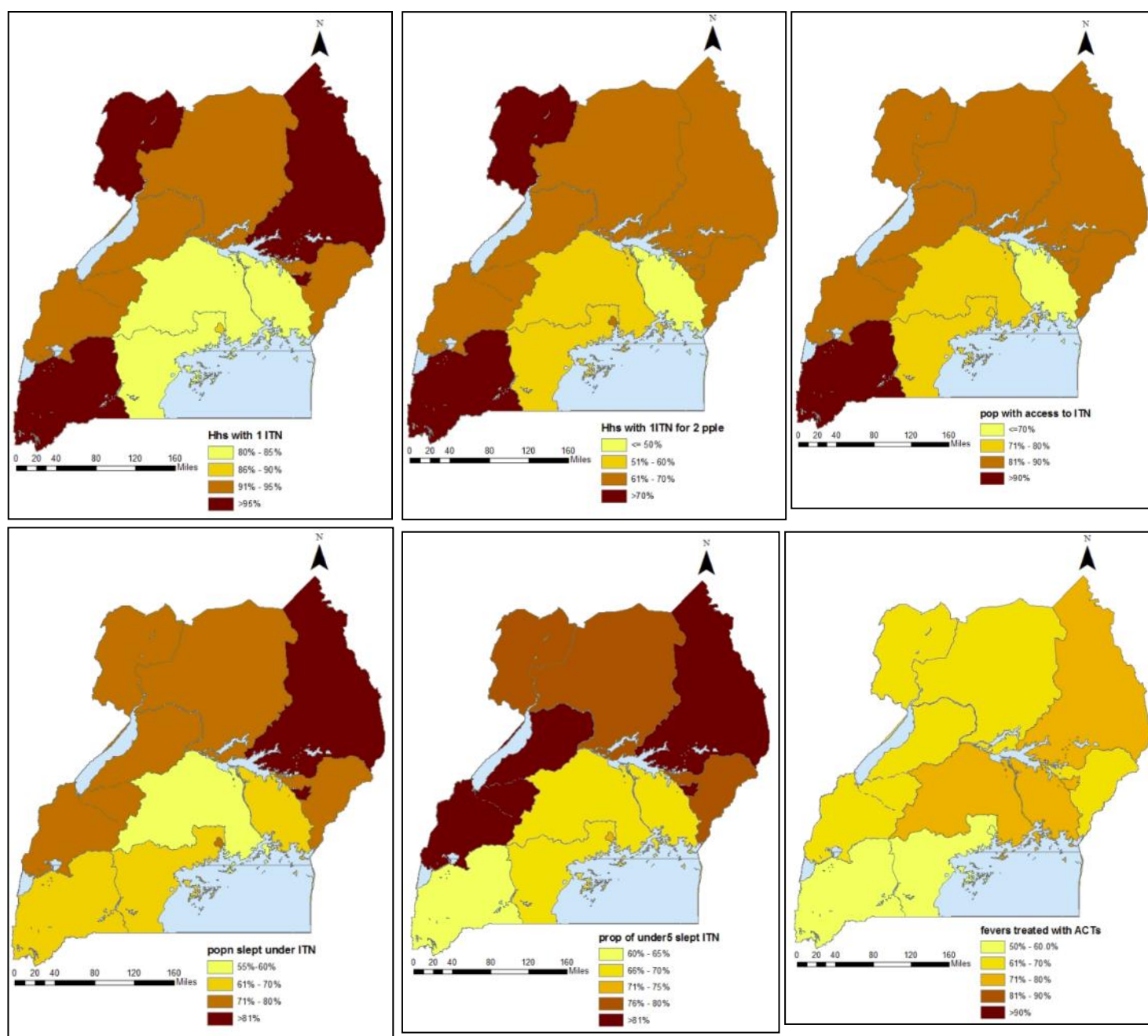

Supplement: S1 Fig — Top; Prop of HHs with 1 ITN (left), Prop of HHs with 1 ITN for two people (middle), Population with access to an ITN (right), Bottom; Prop who slept under an ITN (left), Prop under 5 slept who under ITN (middle), Prop of fevers treated with ACTs(right). (PDF) [file pone.0174948.s001.pdf]

**S2Fig: Distribution of climatic/environmental factors in Uganda in 2014**

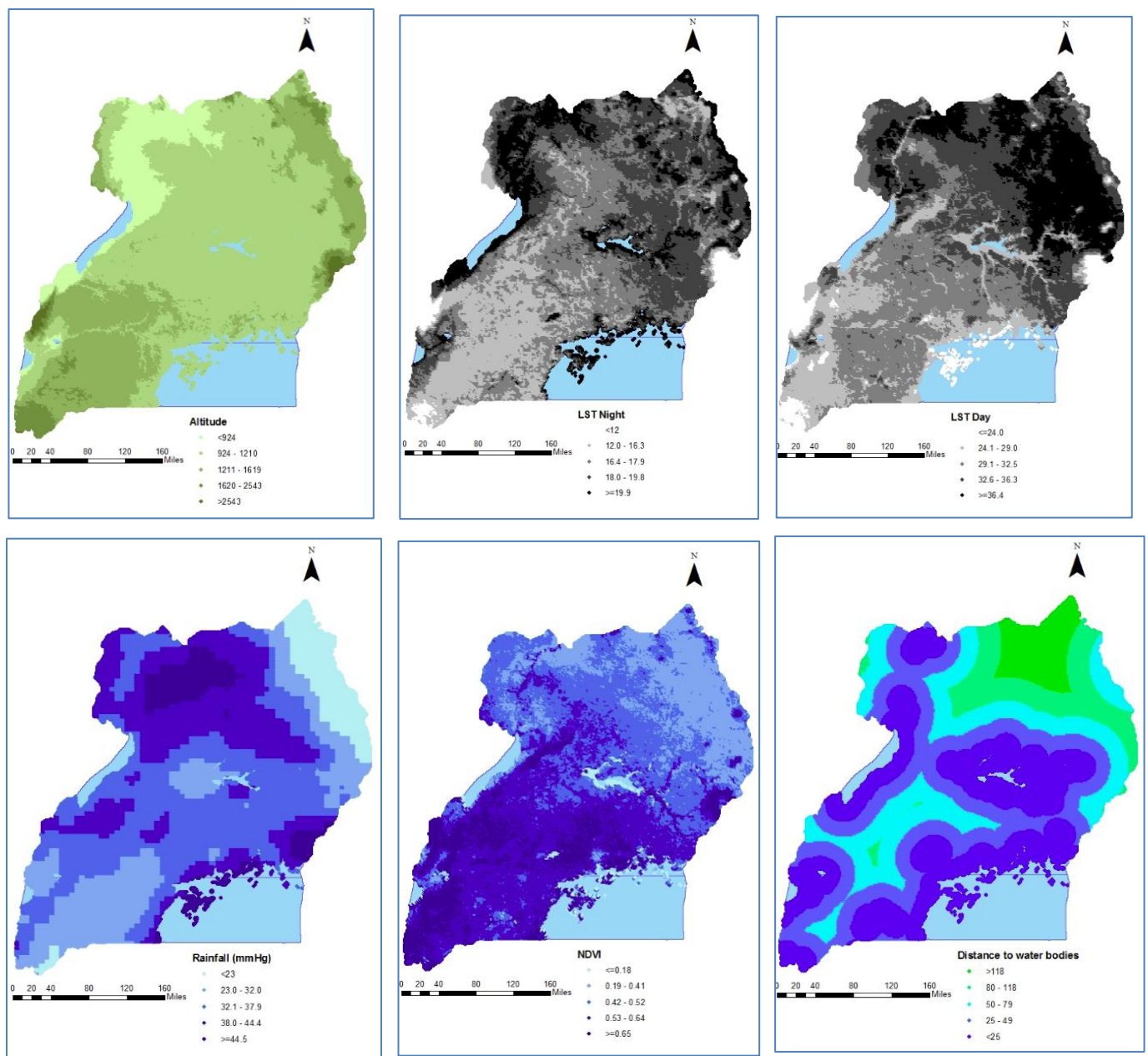

Supplement: S2 Fig — Top; Altitude (left), Night LST (middle), Day LST (right) Bottom; Rainfall (left), NDVI (middle), Distance to water bodies (right). (PDF) [file pone.0174948.s002.pdf]
