# Supplementary material for: Geostatistical modelling of malaria indicator survey data to assess the effects of interventions on the geographical distribution of malaria prevalence in children less than 5 years in Uganda
Source: PLoS One. 2017 Apr 4;12(4):e0174948. doi: 10.1371/journal.pone.0174948 (PMC5380319; doi:10.1371/journal.pone.0174948)
Supplement: S1 Text — (DOCX) [file pone.0174948.s003.docx]

**Statistical modelling details**

**Bernoulli model formulation**

Let $Y_{ij}$ be a binary outcome variable taking value 1 or 0 if a child $i$ at location $S_{j}$ tested positive for malaria. $Y_{ij}$ is assumed to follow a Bernoulli distribution $Y_{ij}\sim Ber(p_{ij})$ and is related to its predictors using a logistic regression model as follows;

$logit\left( p_{ij} \right)=\beta_{0}+\sum_{k=1}^{k} \beta_{k}X_{ij}^{k}+\emptyset_{j}+\omega_{i}$ where $p_{ij}$ is the malaria test result of child $i$ at location $s_{j}$ of having malaria, $\beta={(\beta_{0},\beta_{1},\ldots,\beta_{k})}^{T}$ is the vector of $K$ regression coefficients*.* Employing a geostatistical model formulated in [1], spatial dependence is introduced by adding location-specific random effects $\emptyset_{j}$ at every sampled location $s_{j}$ modeled by a Gaussian process, $\emptyset={(\emptyset_{0},\emptyset_{1},\ldots,\emptyset_{k})}^{T}\sim N(0,\Sigma)$ where Σ is the variance-covariance matrix and each element is defined by an exponential parametric function of the distance $d_{ij}$ between two location $s_{j}$ and $s_{l}$, that is $\sigma^{2}exp(-d_{ij}\rho)$. The parameter $\sigma^{2}$ is the spatial variation and $\rho$ is a smoothing parameter that controls the rate of correlation decay with increasing distance. For exponential correlation function, the range parameter calculated as $\frac{3}{\rho}$ is an estimate of the minimum distance beyond which spatial correlation is negligible. Non-spatial variation is estimated by the random effects $\omega_{i}$, assumed independent and normally distributed with mean 0 and variance $\sigma_{w}^{2}$ . Model fit, parameter estimation and prediction was performed using bayesian formulation and MCMC estimation. Model specification was completed by assigning prior distributions to model parameters. An inverse-gamma prior was assigned for the variance, a gamma distribution for the spatial decay parameter, and non-informative Gaussian distributions for regression coefficients with mean 0 and variance 100.

**Geostatistical variable selection**

To identify the best set of predictor variables and their functional form, bayesian variable selection was done using spike and slab approach [2]. For every predictor $X_{p}$ a categorical indicator parameter $I_{p}$ was introduced to indicate exclusion of the predictor from the model$\left( I_{p}=0 \right)$, inclusion in linear form $\left( I_{p}=1 \right)$ or categorical form $\left( I_{p}=2 \right)$. $I_{p}$ has a probability mass function $\prod_{j=0}^{2} \pi_{j}^{{}_{j}^{\sigma}{{(I}_{p})}}$ where $\pi_{j}$ are the inclusion probability of functional form *j* (i.e. j=0,1,2) such that $\sum_{j=0}^{2} \pi_{j}=1$ and $\sigma_{j}\left( . \right)$ is the Dirac function, $\sigma_{j}\left( I_{p} \right)=f\left( x \right)=\left\{ \begin{aligned} 1, &if I_{p}=j \\ 0, &if I_{p} \neq j \end{aligned} \right.$ . In addition, a spike and slab prior was assumed for the corresponding regression coefficient. For the coefficient $\beta_{p}$ of the predictor in linear form we take $\beta_{p}\sim\delta_{1}\left( I_{p} \right)N\left( 0,\tau_{p}^{2} \right)+\left( 1-\delta_{1}\left( I_{p} \right) \right)N\left( 0,\vartheta_{0}\tau_{p}^{2} \right)$ proposing a non-informative prior for $\beta_{p}$ in case $X_{p}$ is included in the model in linear form (slab) and an informative normal prior shrinking $\beta_{p}$ to zero (spike) if $X_{p}$ is excluded from the model. Similarly, for the coefficient {$\left. \beta_{p,l} \right\}_{l=1,..,L}$ corresponding to the categorical form of with $L$categories, we assume that $\beta_{p,l}\sim\delta_{2}\left( I_{p} \right)N\left( 0,\tau_{p,l}^{2} \right)+\left( 1-\delta_{2} \right)N(0,v_{0}\tau_{p,l}^{2})$ . For the inclusion probabilities, we adopt a non-informative Dirichlet distribution with hyper-parameter $\alpha={(1,1,1)}^{T}$ that is,$\pi={(\pi_{0},\pi_{1},\pi_{2})}^{T}\sim Dirichlet(3,\alpha)$

For addressing correlation between covariates and for speeding up MCMC computation, continuous covariates were standardized.

Model parameters were estimated using MCMC simulation (Gibbs sampling). Starting with some initial values for the parameters, two chains were run initially discarding the first 5000 iterations. Convergence was assessed by Gelman and Rubin diagnostic [3] and kernel density plots were used to assess for convergence of the chains.

**Estimating the effect of intervention at regional level**

The model above was extended to include intervention coverage effects with spatially varying coefficients, that is, $ogit\left( p_{ij} \right)=\beta_{0}+\sum_{k=1}^{k} \beta_{k}X_{ij}^{(k)}+\sum_{m=1}^{M} b_{mj}Z_{i}^{\left( m \right)}\left( A_{j} \right)+\emptyset_{j}$, where $Z_{i}^{\left( m \right)}(A_{j})$ is the $m$ intervention coverage aggregated over region $A_{j}$ of the $s_{j}$ location, $b_{mj}$ is the corresponding spatially varying coefficient (i.e. intervention effect at $A_{j}$ region) and $M$ is the number of spatially varying interventions. Gaussian conditional autoregressive (CAR) prior distributions were assumed for the $b_{m}$, that is $b_{m}\sim N(b_{m0}1,\sum_{m})$ where $b_{m0}$ is the global effect of the $m$ intervention at country level and $\sum_{m}^{-1}=\sigma_{m}^{-2}(D-W)$, $D$ is a diagonal matrix with elements the sum of the neighbours of each region, $W$ is a proximity matrix.
